# Cerebral microdialysis reflects the neuroprotective effect of fractionated plasma separation and adsorption in acute liver failure better and earlier than intracranial pressure: a controlled study in pigs

**DOI:** 10.1186/1471-230X-13-98

**Published:** 2013-06-08

**Authors:** Josef Prazak, Eva Laszikova, Tomas Pantoflicek, Ondrej Ryska, Eva Koblihova, Miroslav Ryska

**Affiliations:** 1 Department of Anaesthesiology and Intensive Care, University Hospital, Basel, Switzerland; 2 Department of Anaesthesiology, Resuscitation and Intensive Care, Second Faculty of Medicine, Charles University, Prague, Czech Republic; 3 Department of Anaesthesiology and Resuscitation, First Faculty of Medicine and Central Military Hospital, Prague, Czech Republic; 4 Department of Surgery, Second Faculty of Medicine, Charles University and Central Military Hospital, Prague, Czech Republic

**Keywords:** Cerebral microdialysis, Intracranial pressure, Acute liver failure, Fractionated plasma separation and adsorption

## Abstract

**Background:**

Cerebral edema is a well-recognized and potentially fatal complication of acute liver failure (ALF). The effectiveness of treatments that address intracranial hypertension is generally assessed by measuring intracranial pressure (ICP). The aim of this study was to determine the role of cerebral microdialysis in monitoring the efficacy of fractionated plasma separation and adsorption (FPSA) treatment for ALF. We hypothesized that in ALF cerebral microdialysis reflects the benefits of FPSA treatment on cerebral edema before ICP.

**Methods:**

A surgical resection model of ALF was used in 21 pigs. We measured plasma ammonia concentration, brain concentrations of glucose, lactate, pyruvate, glutamate and glutamine, and ICP. Animals were randomized into three groups: in one group eight animals received 6 hours of FPSA treatment 2 hours after induction of ALF; in another group 10 animals received supportive treatment for ALF only; and in the final group three underwent sham surgery.

**Results:**

The ICP was significantly higher in the ALF group than in the FPSA group 9 hours after surgery. The lactate/pyruvate (L/P) ratio was significantly lower in the FPSA group than the ALF group 5 hours after surgery, before any significant difference in ICP was detected. Indeed, significant changes in the L/P ratio could be observed within 1 hour of treatment. Glutamine levels were significantly lower in the FPSA group than the ALF group between 6 hours and 10 hours after surgery.

**Conclusions:**

Brain lactate/pyruvate ratio and concentration of glutamine measured by cerebral microdialysis reflected the beneficial effects of FPSA treatment on cerebral metabolism more precisely and rapidly than ICP in pigs with fulminant ALF. The role of glutamine as a marker of the efficacy of FPSA treatment for ALF appears promising, but needs further evaluation.

## Background

Hepatic encephalopathy, causing increased intracranial pressure (ICP) and brain injury, is a well-known and potentially fatal complication of acute liver failure (ALF) [1]. Orthotopic liver transplantation remains the only treatment for fulminant ALF that significantly improves the chances of survival [2]. A variety of approaches may be taken to meet homeostatic needs during the critical period when a donor organ is sought (bridging therapy). Supportive therapy using fractionated plasma separation and adsorption (FPSA) appears to have a beneficial effect on intracranial hypertension [3]. The efficacy of FPSA therapy in treatment of ALF-related brain injury is usually monitored by measuring ICP. Recently, the potential of using cerebral microdialysis to monitor cerebral metabolism has been explored in patients with ALF [4], as has the effect of FPSA on the intracerebral oxidative metabolism in ALF [5]. To date, one clinical trial has reported that FPSA had no effect on brain metabolism as measured by the lactate to pyruvate (L/P) ratio, although the patient cohort was small and comprised patients with different etiologies of ALF [5]. There have been no studies in large animal models.

The objective of this trial was to evaluate the role of cerebral microdialysis in monitoring FPSA therapy in a well-defined pig model of ALF, and compare it with intracerebral pressure (ICP), a parameter used more frequently in clinical practice. We hypothesized that microdialysis parameters would reflect the benefit of FPSA treatment on brain injury in ALF earlier than ICP. Our secondary outcome measure was to examine the influence of intracerebral glutamine concentration as a marker of the therapeutic efficacy of FPSA.

## Methods

The surgical resection model of ALF was undertaken in 23 pigs weighing 35–40 kg. This modified model [6] combined 70% resection of the hepatic parenchyma and 20 min total venous hepatic occlusion. Two hours after surgery, 20 animals were randomized into a control group (ALF group) and a group treated by FPSA (FPSA group). The animals in the FPSA group were connected to the elimination device (Prometheus system, Fresenius Medical Care AG, Germany), as described below, for 6 hours of treatment 2 hours after surgery had been completed [7]. In the SHAM group (three animals) a laparotomy alone was performed. Twelve hours after surgery, all animals were euthanized by a lethal dose of thiopental (sodium pentothal, Abbott Laboratories, UK) and 7.45% potassium chloride (Braun). In the FPSA group, two animals died postoperatively as a consequence uncontrollable hemorrhage. In total, 21 animals (10 in the control group, 8 in the FPSA treated group and 3 in the SHAM group) completed the study.

### Anesthesia

Animals were premedicated with intramuscular ketamine 10 mg/kg (Narkamon, Léčiva, Prague, Czech Republic), atropine 0.01 mg/kg (Atropin Biotika, Hoechst-Biotika, Martin, Slovakia), azaperone 4 mg/kg (Stresnil, Janssen Pharmaceutical, Beerse, Belgium) and medetomidine 25 μg/kg (Domitor, Pfizer, NY, USA) 20 minutes before induction. General anesthesia was induced with intravenous fentanyl 4 μg/kg (Fentanyl Torrex, Torrex Chiesi, Vienna, Austria) and etomidate 0.3 mg/kg (Hypnomidate, Janssen Pharmaceutica); then the trachea was intubated. Positive pressure ventilation was maintained (Siemens Servo 900 C ventilator, Siemens, Elema, Sweden) in volume controlled mode, with a fraction of inspired oxygen of 0.4, positive end expiratory pressure of 5 cm H_2_O, frequency 16 breaths/min, tidal volume 6–8 mL/kg so as to achieve normocapnia (paCO_2_ 4.6–5.3 kPa). Maintenance anesthesia was provided using isoflurane (Forane, Abbott Laboratories, UK) with an intravenous infusion of 6–10 μg/kg/h fentanyl. Neuromuscular blockade was achieved by means of boluses of 0.02 mg/kg pipecuronium bromide (Arduan, Gedeon Richter, Budapest, Hungary) given throughout surgery. Crystalloid and colloid solutions were used to support the circulation and address hemorrhage; an infusion of norepinephrine (Léčiva) was administered when needed. The protocol for anesthesia and intravascular volume therapy was the same in each experimental group. Augmentin 1.2 g (amoxicillin and clavulanic acid, SmithKline Beecham Pharmaceuticals, London, UK) and famotidine 20 mg (Quamatel, Gedeon Richter) were administered as antibiotic prophylaxis and to suppress gastric acid secretion, respectively. Catheters were inserted into the femoral artery and vein under direct surgical vision to allow connection of the FPSA extracorporeal device, and to allow invasive blood pressure monitoring and collection of blood samples. The internal jugular vein was identified surgically, and a thermodilution pulmonary artery catheter (7F Arrow, PA, USA) was positioned to monitor hemodynamic variables.

### Postoperative management

After liver resection, mechanical ventilation was changed to pressure controlled mode, to achieve normocapnia with a tidal volume of 6 to 8 mL/kg. The body temperature was maintained between 36.5°C and 38.0°C using a forced-air warming system (WarmAir, CSZ Medical). Subsequent sedation and analgesia were provided by infusions of propofol 6 mg/kg/h (Fresenius Kabi, Bad Homburg, Germany) and fentanyl 2 mg/kg/h. Crystalloid and colloid solutions were given as needed. Blood glucose concentration was maintained above 4.5 mmol/l using a continuous infusion of 40% glucose solution (Braun). The circulation was supported when necessary with a norepinephrine infusion to maintain a mean arterial pressure >65 mmHg.

### Microdialysis and ICP monitoring

Microdialysis and ICP monitoring was initiated within the first postoperative hour. Two burr holes were created over the right frontal bone under sterile conditions. The dura mater was punctured, and the microdialysis membrane and ICP sensor were placed separately into the frontal lobe. To prevent leakage of cerebrospinal fluid (CSF), the burr holes were sealed with bone wax. The CMA70 microdialysis catheter (CMA Solna Sweden) consists of a thin, double lumen, 60 mm plastic tube with a 10 mm semipermeable 20 kDa membrane at the tip. After insertion into the brain tissue, the catheter was connected to a microinfusion pump (CMA/106 Microinjection pump; CMA Microdialysis AB, Stockholm, Sweden) and continuously perfused with artificial CSF at a rate of 0.3 μL/min. The dialysate was collected in microvials, each containing approximately 18 μL aliquots for further analysis (CMA 600 Microdialysis Analyzer, CMA Microdialysis). The analyzer measures glucose, lactate, pyruvate and glutamate concentrations in each sample, with >99% recovery when using a buffer of known concentration at the selected perfusion rate [8,9]. We collected dialysate samples every 60 min for analysis; the first sample was collected during the second postoperative hour. Specimens were frozen at −80°C for later analysis of glutamine concentration using a Perkin-Elmer reverse-phase high pressure liquid chromatography system with fluorescence detection and precolumn *o*-phthalaldehyde derivatization [10]. ICP monitoring was performed using an intraparenchymal sensor (Codan Microsensor, Johnson and Johnson, USA).

### FPSA treatment

The Prometheus extracorporeal system was primed with 0.9% NaCl, prior to its connection to the venous dialysis catheter in the right femoral vein. The device eliminates water-soluble and protein-bound toxins and breakdown products of metabolism. In the FPSA circuit, venous blood passes through a separator with a pore size of 250 kDa (AlbuFlow, Fresenius). The separated plasma than passes through a neutral resin absorbent column (Prometh01, Fresenius) and an anion exchange resin absorber (Prometh02, Fresenius) to remove albumin-bound toxins. The plasma phase is then returned to the filter and dialyzed as whole blood in a high flow dialyzer (F60S, Fresenius) to remove water-soluble toxins [11] before being returned to the femoral vein. The treatment was performed with a blood flow of 200 mL/min and a plasma flow of 300 mL/min. Sodium citrate was used as an anticoagulant. The dose of sodium citrate was determined according to serum ionized calcium concentration.

### Statistical analysis

Regression models were constructed separately for each parameter in each group of observations. The models consisted of a polynomial of at most fifths degree with fitted intercepts; terms were calculated using the least squares method. The model was developed by initially including all terms, then the term with the lowest significance was sequentially removed until all terms demonstrated significance on an α = 5% level. The intercept was not removed. The first-degree term was retained in models where none of the terms demonstrated significance. The fits were calculated for the chosen time points, including the standard error, and 95% confidence intervals (95%CI). In addition, analysis of variance (ANOVA) was undertaken using the same model. For each given time point, a two-sample t-test was undertaken to examine the following hypotheses: H0, the difference between fits was not significant; H1, the difference between fits was significant. The t-test was calculated using the following parameters: mean difference (difference between the compared fits); degrees of freedom (degrees of freedom corresponding to the error term in ANOVA, i.e. the number of observations minus the number of terms included in the regression model); and standard deviation (standard error of the fit multiplied by the square root of [degrees of freedom +1]). A p-value (the smallest level to conclude significance) was calculated from the test statistic T. The acceptability of the alternative hypothesis was evaluated on an α = 5% level (p <0.05). The regression model was developed in Q-DAS Destra v.10 (Q-DAS Asset GmbH & Co. KG, Weinheim, Germany). The fits and associated parameters were calculated in Minitab 16.2.2. The t-test was performed and data plotted using Microsoft MS Excel 2010.

Preoperative treatment, surgery and postoperative care were undertaken according to the protection against cruelty to animals, law no. 312/2008 Coll., and the protection, breeding and the use of experimental animals, decree no. 207/2004 Coll. The study was approved by the experts and ethics committee of the Institute of Clinical and Experimental Medicine in Prague, Czech Republic.

## Results

### Intracranial pressure

**Figure 1:**
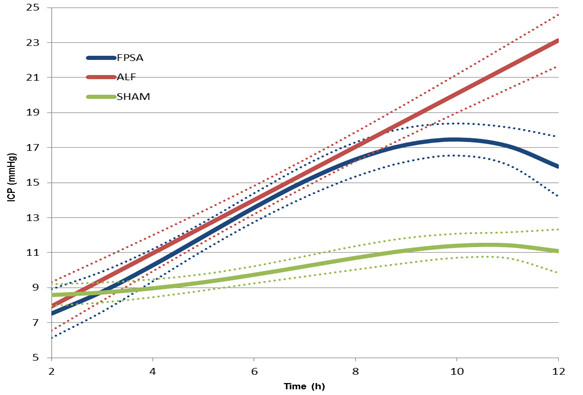
**Intracranial pressure (ICP) following fractionated plasma separation and adsorption treatment (2–8 h) (curve fits with 95% confidence intervals shown).** Abbreviations: FPSA, fractionated plasma separation and adsorption; ALF, acute liver failure; SHAM, sham-operated controls.

The ICP was significantly higher (p <0.05) in the ALF group between hours 9–12 when compared with the FPSA-treated group. After 5 hours, ICP was significantly higher (p <0.05) in the ALF and FPSA groups compared with the SHAM group (Figure 1, Table 1); however, the magnitude of the increase was significantly smaller (p <0.05) in the FPSA than the ALF group.

**Table 1:** Intracranial pressure (ICP), lactate/pyruvate (L/P) ratio, extracellular brain glutamine, extracellular brain glutamate, extracellular brain glucose and arterial ammonia following fractionated plasma separation and adsorption (FPSA) treatment

	**Tme (h)**	**2**	**3**	**4**	**5**	**6**	**7**	**8**	**9**	**10**	**11**	**12**
	**FPSA**	**7,53**	**8,77**	**10,29**	**11,93**	**13,58**	**15,09**	**16,33**	**17,17**	**17,47**	**17,10**	**15,92**
	*SE*	*0,70*	*0,58*	*0,46*	*0,40*	*0,41*	*0,46*	*0,49*	*0,49*	*0,46*	*0,54*	*0,85*
**ICP**	**ALF**	**7,94**	**9,46**	**10,98**	**12,49**	**14,01**	**15,53**	**17,05**	**18,57**	**20,09**	**21,61**	**23,13**
(mmHg)	*SE*	*0,70*	*0,60*	*0,52*	*0,45*	*0,41*	*0,40*	*0,42*	*0,48*	*0,55*	*0,64*	*0,74*
	**SHAM**	**8,58**	**8,73**	**8,97**	**9,31**	**9,74**	**10,22**	**10,70**	**11,12**	**11,40**	**11,43**	**11,09**
	*SE*	*0,30*	*0,28*	*0,25*	*0,23*	*0,24*	*0,28*	*0,33*	*0,35*	*0,34*	*0,36*	*0,61*
	*FPSAxALF*	*0,68*	*0,42*	*0,33*	*0,36*	*0,46*	*0,46*	*0,26*	*0,04*	*< 0,01*	*< 0,01*	*< 0,01*
***p values***	*FPSAxSHAM*	*0,33*	*0,96*	*0,07*	*< 0,01*	*< 0,01*	*< 0,01*	*< 0,01*	*< 0,01*	*< 0,01*	*< 0,01*	*< 0,01*
	*ALFxSHAM*	*0,58*	*0,47*	*0,02*	*< 0,01*	*< 0,01*	*< 0,01*	*< 0,01*	*< 0,01*	*< 0,01*	*< 0,01*	*< 0,01*
	**FPSA**	**32,29**	**25,81**	**21,03**	**17,71**	**15,63**	**14,54**	**14,19**	**14,36**	**14,80**	**15,27**	**15,54**
	*SE*	*2,98*	*1,70*	*1,62*	*1,65*	*1,54*	*1,49*	*1,63*	*1,79*	*1,73*	*1,69*	*2,83*
**L/P ratio**	**ALF**	**22,19**	**22,00**	**21,81**	**21,62**	**21,43**	**21,24**	**21,05**	**20,86**	**20,67**	**20,47**	**20,28**
	*SE*	*1,06*	*0,92*	*0,80*	*0,70*	*0,63*	*0,61*	*0,63*	*0,70*	*0,80*	*0,92*	*1,06*
	**SHAM**	**9,88**	**9,92**	**9,96**	**10,00**	**10,03**	**10,07**	**10,11**	**10,15**	**10,19**	**10,23**	**10,26**
	*SE*	*0,88*	*0,76*	*0,66*	*0,58*	*0,54*	*0,54*	*0,58*	*0,66*	*0,76*	*0,88*	*1,01*
	FPSAxALF	<0,05	0,40	0,64	0,02	<0,01	<0,01	<0,01	<0,01	<0,01	<0,01	0,08
***p values***	FPSAxSHAM	<0,01	<0,01	<0,01	<0,01	0,02	0,05	0,10	0,12	0,08	0,05	0,05
	ALFxSHAM	<0,01	<0,01	<0,01	<0,01	<0,01	<0,01	<0,01	<0,01	<0,01	<0,01	<0,01
	**FPSA**	**0,66**	**0,92**	**1,08**	**1,17**	**1,20**	**1,19**	**1,20**	**1,27**	**1,48**	**1,90**	**2,66**
	*SE*	*0,16*	*0,12*	*0,12*	*0,12*	*0,11*	*0,11*	*0,12*	*0,13*	*0,13*	*0,15*	*0,29*
**Extracellular brain**	**ALF**	**1,08**	**1,23**	**1,37**	**1,52**	**1,66**	**1,81**	**1,95**	**2,10**	**2,24**	**2,39**	**2,53**
**glutamine**	*SE*	*0,30*	*0,26*	*0,22*	*0,19*	*0,16*	*0,15*	*0,16*	*0,18*	*0,21*	*0,25*	*0,29*
(μmol/L)	**SHAM**	**0,07**	**0,12**	**0,19**	**0,26**	**0,33**	**0,40**	**0,46**	**0,50**	**0,52**	**0,52**	**0,48**
	*SE*	*0,09*	*0,08*	*0,06*	*0,04*	*0,03*	*0,04*	*0,04*	*0,04*	*0,04*	*0,04*	*0,07*
	FPSAxALF	0,21	0,27	0,25	0,12	0,02	<0,01	<0,01	<0,01	<0,01	0,09	0,76
*p*	FPSAxSHAM	0,05	<0,01	<0,01	<0,01	<0,01	<0,01	<0,01	<0,01	<0,01	<0,01	<0,01
	ALFxSHAM	0,07	0,02	<0,01	<0,01	<0,01	<0,01	<0,01	<0,01	<0,01	<0,01	<0,01
	**FPSA**	**19,82**	**16,22**	**13,20**	**10,77**	**8,93**	**7,69**	**7,03**	**6,97**	**7,49**	**8,61**	**10,32**
	*SE*	*3,24*	*2,20*	*1,70*	*1,69*	*1,85*	*1,94*	*1,89*	*1,77*	*1,76*	*2,17*	*3,12*
**Extracellular brain glutamate**	**ALF**	**29,65**	**28,83**	**28,01**	**27,19**	**26,38**	**25,56**	**24,74**	**23,92**	**23,11**	**22,29**	**21,47**
(μmol/L)	*SE*	*6,50*	*5,58*	*4,74*	*4,04*	*3,55*	*3,37*	*3,55*	*4,04*	*4,74*	*5,58*	*6,50*
	**SHAM**	**12,54**	**12,03**	**11,51**	**11,00**	**10,48**	**9,97**	**9,46**	**8,94**	**8,43**	**7,92**	**7,40**
	*SE*	*2,03*	*1,76*	*1,52*	*1,33*	*1,21*	*1,20*	*1,28*	*1,45*	*1,68*	*1,94*	*2,23*
	FPSAxALF	0,22	0,06	0,01	<0,01	<0,01	<0,01	<0,01	<0,01	0,01	0,04	0,16
***p values***	FPSAxSHAM	0,15	0,23	0,54	0,93	0,59	0,45	0,41	0,48	0,74	0,84	0,55
	ALFxSHAM	0,12	0,08	0,04	0,02	0,01	0,01	0,01	0,03	0,07	0,13	0,20
	**FPSA**	**1,71**	**1,69**	**1,66**	**1,64**	**1,62**	**1,59**	**1,57**	**1,54**	**1,52**	**1,49**	**1,47**
	*SE*	*0,27*	*0,23*	*0,20*	*0,17*	*0,15*	*0,15*	*0,16*	*0,18*	*0,21*	*0,24*	*0,28*
**Extracellular brain glucose**	**ALF**	**1,52**	**1,50**	**1,46**	**1,41**	**1,35**	**1,27**	**1,19**	**1,09**	**0,99**	**0,87**	**0,74**
(mmol/L)	*SE*	*0,12*	*0,12*	*0,11*	*0,10*	*0,09*	*0,08*	*0,08*	*0,09*	*0,10*	*0,12*	*0,15*
	**SHAM**	**2,45**	**2,42**	**2,37**	**2,32**	**2,25**	**2,16**	**2,07**	**1,96**	**1,83**	**1,70**	**1,55**
	*SE*	*0,13*	*0,13*	*0,12*	*0,11*	*0,10*	*0,09*	*0,09*	*0,11*	*0,13*	*0,16*	*0,20*
	FPSAxALF	0,49	0,42	0,33	0,21	0,11	0,05	0,02	0,01	0,01	0,01	0,02
*p values*	FPSAxSHAM	0,09	0,05	0,03	0,02	0,01	0,02	0,05	0,15	0,34	0,60	0,86
	ALFxSHAM	<0,01	<0,01	<0,01	<0,01	<0,01	<0,01	<0,01	<0,01	<0,01	<0,01	0,01
	**FPSA**	**243,1**	**223,8**	**220,5**	**236,6**	**263,7**	**287,1**	**293,8**	**277,7**	**247,3**	**232**	**288,3**
	*SE*	*28,96*	*29,04*	*26,88*	*25,65*	*29,04*	*31,18*	*28,09*	*31,05*	*47,24*	*52,05*	*33,53*
**Arterial ammonia**	**ALF**	**235,2**	**271,5**	**302,3**	**327,7**	**347,6**	**362**	**371**	**374,5**	**372,6**	**365,2**	**352,3**
(μmol/L)	*SE*	*24,2*	*26,39*	*28,48*	*29,67*	*29,68*	*28,61*	*26,94*	*25,77*	*26,86*	*31,84*	*41,05*
	**SHAM**	**31,84**	**32,7**	**33,57**	**34,44**	**35,31**	**36,17**	**37,04**	**37,91**	**38,78**	**39n65**	**40n51**
	*SE*	*3,78*	*3,48*	*3,29*	*3,24*	*3,33*	*3,57*	*3,91*	*4,34*	*4,82*	*5,36*	*5,93*
	FPSAxALF	0,83	0,23	0,05	0,03	0,05	0,09	0,06	0,02	0,02	0,02	0,26
***p values***	FPSAxSHAM	<0,01	<0,01	<0,01	<0,01	<0,01	<0,01	<0,01	<0,01	<0,01	<0,01	<0,01
	ALFxSHAM	<0,01	<0,01	<0,01	<0,01	<0,01	<0,01	<0,01	<0,01	<0,01	<0,01	<0,01

*SE* standard error.

### Microdialysis parameters

**Figure 2:**
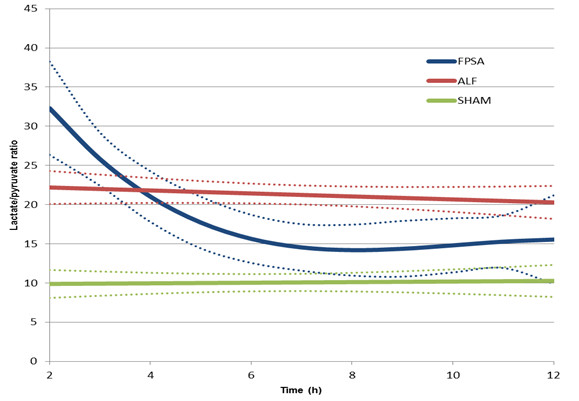
**Extracellular brain lactate/pyruvate ratio following fractionated plasma separation and adsorption treatment (2–8 h) (curve fits with 95% confidence intervals shown).** Abbreviations: FPSA, fractionated plasma separation and adsorption; ALF, acute liver failure; SHAM, sham-operated controls.

**Figure 3:**
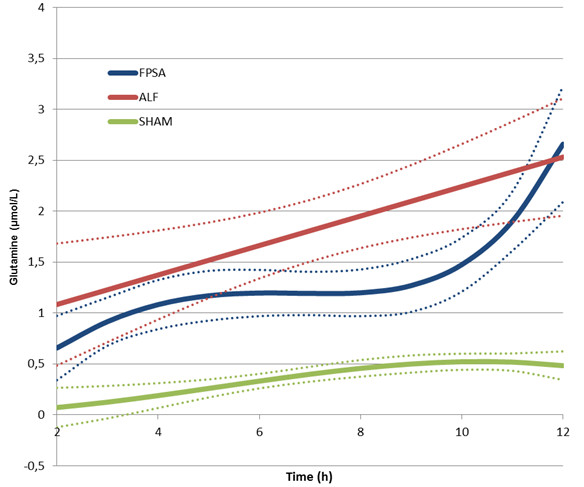
**Extracellular brain glutamine concentration following fractionated plasma separation and adsorption treatment (2–8 h) (curve fits with 95% confidence intervals shown).** Abbreviations: FPSA, fractionated plasma separation and adsorption; ALF, acute liver failure; SHAM, sham-operated controls.

**Figure 4:**
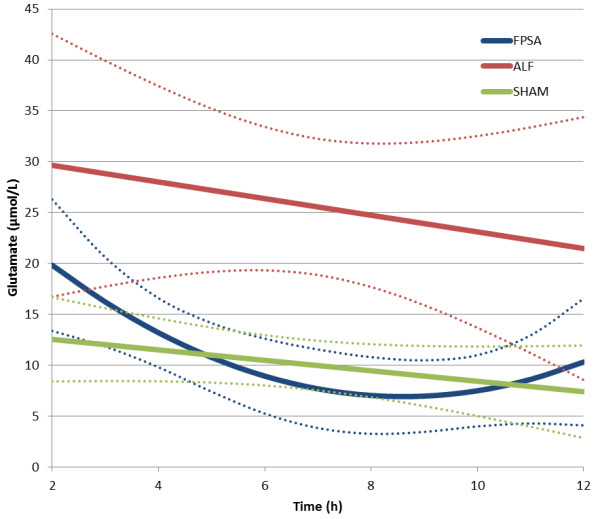
**Extracellular brain glutamate concentration following fractionated plasma separation and adsorption treatment (2–8 h) (curve fits with 95% confidence intervals shown).** Abbreviations: FPSA, fractionated plasma separation and adsorption; ALF, acute liver failure; SHAM, sham-operated controls.

**Figure 5:**
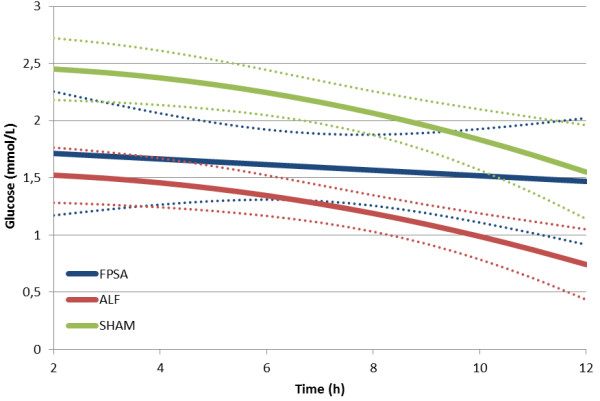
**Extracellular brain glucose concentration following fractionated plasma separation and adsorption treatment (2–8 h) (curve fits with 95% confidence intervals shown).** Abbreviations: FPSA, fractionated plasma separation and adsorption; ALF, acute liver failure; SHAM, sham-operated controls.

There was a significant decrease in the lactate to pyruvate ratio (L/P ratio) in the FPSA group, but not in the ALF or SHAM groups. The L/P ratio was significantly higher in the ALF group than the SHAM group between 2 hours and 12 hours after surgery from (p <0.01), and significantly lower in the FPSA group than the ALF group between 5 hours and 11 hours after surgery (p <0.05). Comparing the FPSA and the SHAM groups, The L/P ratio was significantly higher in the FPSA group than the SHAM group between 2 hours and 7 hours, and from 11 hours after surgery (p <0.05, Figure 2, Table 1). The extracellular brain glutamine concentration was significantly higher in both the ALF and FPSA groups compared with the SHAM group from 2 hours to 12 hours after surgery, and 3 hours to 12 hours after surgery, respectively (p <0.05; p <0.02). Glutamine concentrations were significantly lower in the FPSA group compared with the ALF group from 6 hours to 10 hours after surgery (p <0.05). The increase in glutamine levels was significantly higher (p <0.05) in the ALF than in the FPSA group (Figure 3, Table 1). Extracellular brain glutamate concentrations were significantly higher in the ALF group than the FPSA group between 4 hours and 11 hours after surgery (p <0.05), and the SHAM group between 4 hours and 9 hours after surgery (p <0.05). Glutamate concentration was higher in the FPSA group than the SHAM group between 2 hours and 4 hours after surgery, lower between 5 hours and 10 hours, and higher again between 11 hours and 12 hours after surgery but the difference was not significant (Figure 4, Table 1). The extracellular brain glucose concentration was significantly higher in the SHAM group than either the ALF or the FPSA groups 2 hours to 12 hours after surgery and 3 hours to 7 hours after surgery, respectively (p <0.05 for both). The extracellular glucose level was significantly higher (p <0.05) in the FPSA group compared with the ALF group from 7 hours after surgery (Figure 5, Table 1).

### Plasma ammonia

**Figure 6:**
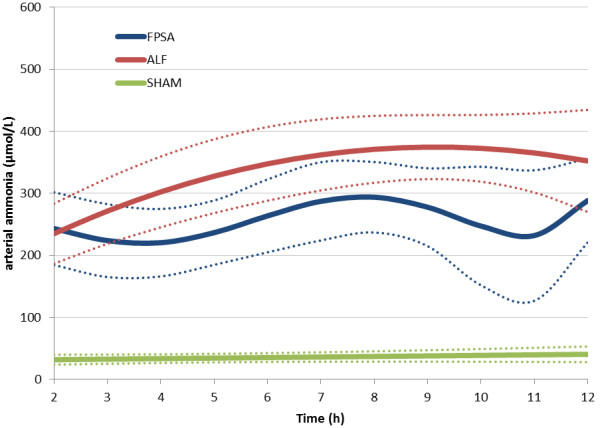
**Arterial ammonia concentration following fractionated plasma separation and adsorption treatment (2–8 h) (curve fits with 95% confidence intervals shown).** Abbreviations: FPSA, fractionated plasma separation and adsorption; ALF, acute liver failure; SHAM, sham-operated controls.

The plasma ammonia concentration was significantly higher in the FPSA and ALF groups than the SHAM group, and higher in the ALF group than the FPSA group between 5 hours and 6 hours, and between 9 hours and 11 hours after surgery (p <0.05) (Figure 6, Table 1). Compared with baseline values, plasma ammonia was significantly lower in the FPSA group than the ALF group from 4 hours. In the SHAM and FPSA groups, plasma ammonia concentrations were not significantly elevated, but there was a significant increase (p <0.05) in the ALF group.

## Discussion

ALF carries a high mortality rate [12], frequently precipitating multiorgan failure (MOF) regardless of the cause. The onset of ALF is accompanied by hepatic encephalopathy and cerebral edema, leading to a rise in ICP. Intracranial hypertension, brain injury and MOF are the most common causes of death in ALF [13]. Intracranial hypertension in ALF is caused by the swelling of brain cells, particularly astrocytes [14], as a consequence of the accumulation of toxic metabolites that have not been cleared by the liver, and by cerebral hyperemia, caused by disruption of the autoregulation of cerebral blood flow [15]. Liver transplantation may afford the only chance of a cure in many patients with fulminant ALF. Various bridging therapy procedures have been proposed to overcome the critical period before transplantation. The FPSA method attenuates the rise in ICP caused by ALF.

Cerebral microdialysis allows accurate monitoring of the contents of the extracellular space [16]. The L/P ratio is a well-recognized marker of the redox state of the tissue and an indicator of tissue ischemia [17], and in the brain reflects the metabolic state of astrocytes. Other markers of cellular homeostasis routinely detected and analyzed by microdialysis can also provide further information about pathological processes in brain tissue. The amino acid glutamate, the main excitatory neurotransmitter released upon neuronal excitation, is considered to be an indirect marker of neuronal tissue distress.

We found that the L/P ratio decreased significantly and almost immediately after the start of FPSA treatment, reflecting a return to the normal metabolic state of the brain, although it is notable that the ratio was high immediately after surgery. Presumably this reflects the extent of surgical stress and hepatic impairment, although this was the same for animals in the FPSA and ALF groups. Within 6 hours, the L/P ratio in the FPSA group had fallen to match that of the SHAM controls, but there was no decrease in the ALF group. Later, between 10 and 12 hours, the L/P ratio in the FPSA group rose again until it was similar to the ALF group, suggesting that the benefit of FPSA treatment wanes over time. Significant differences in the L/P ratio were seen between the FPSA and ALF groups 1 hour after treatment, although surprisingly it was higher in the FPSA group. It is probable that the first specimens contained dialysate collected during equilibration just after insertion of the ICP and microdialysis catheters, and that our conclusions about early observations may be limited by early direct trauma to the brain during placement of the devices [18].

In contrast to the clinical trial studying the effect of FPSA on oxidative metabolism in patients with ALF conducted by Bjerring and colleagues [5], we found a significant difference between the treated and control groups and a significant decrease in the L/P ratio in the treated group. This could be explained by our use of a well-defined and predictable animal model with a control group, whereas the etiology of ALF varied in the cohort of patients enrolled in the clinical trial. We were also able to control the timing of sampling and the FPSA treatment in relation to the induction of ALF precisely, which is more challenging in clinical practice, and to collect microdialysate samples hourly rather than before and after treatment. The reporting of the clinical trial is also unclear about the definition of completion of treatment, and whether some of the dialysate might have been collected before FPSA was complete as the perfusion rate used equated to a sampling period exceeding 5 hours. As the L/P ratio changes rapidly during FPSA treatment, our results are likely to be more informative.

The ICP increased significantly in the ALF and FPSA groups, and was significantly higher than in the SHAM group from 5 hours onwards. The ICP was significantly higher in the ALF group compared with the FPSA group from the ninth hour. The rise in ICP observed in the FPSA group, even if it was significantly less than the ALF group, suggests a multiple causes for the intracranial hypertension associated with ALF [14], which can only be partially ameliorated by the supporting toxin-eliminating method. Our findings support the conclusions of previous studies and confirm the efficacy of toxin elimination in as a treatment for intracranial hypertension in ALF [3]*.* The most significant difference between the ICP measured in the FPSA and control groups was observed 9 hours after surgery (7 hours after initiation of FPSA therapy). Thus, the L/P ratio appears to reflect the positive influence of FPSA treatment on cerebral metabolism more promptly than ICP.

ICP tends to change gradually, but substantial and significant changes in L/P ratio were evident almost immediately after FPSA treatment. This suggests that FPSA is having an almost immediate beneficial effect on cerebral metabolism, and that L/P ratio may be a more useful means of guiding interventions in clinical practice [19].

Correlations between intracerebral glutamine concentration, ICP and arterial ammonia concentration have been reported in clinical studies of ALF [20]. Ammonia crosses the blood–brain barrier easily and increases brain glutamine levels by accelerating its synthesis, predominantly by astrocytes [21]. Intracellular accumulation of glutamine in the astrocytes causes osmotic cell swelling and high ICP [22]. *In vitro* studies suggest that glutamine might also cause metabolic failure within mitochondria, having been transported into mitochondria where it is metabolized to glutamate and ammonia [23]. This in turn triggers mitochondrial permeability transition and gives rise to free radical production, both of which contribute to astrocyte swelling and brain edema [24]. Elevated intracellular glutamine concentration is thought to be one of the most important causes of mitochondrial distress and intracellular hypoxia in ALF. Our data clearly show a positive influence of FPSA on the cerebral accumulation of glutamine, and support the hypothesis that accumulation of cerebral glutamine contributes to intracranial hypertension in ALF. Moreover, our findings are consistent with those in patients with ALF in which correlations between cerebral glutamine, ICP and the L/P ratio were evident [25]. Significantly lower concentrations of glutamine were observed in the FPSA group compared with the ALF group 6 hours after surgery, 3 hours before significant differences in ICP became evident. However, glutamine assays are technically difficult and bedside testing is not readily available; further research is needed to establish the clinical utility of brain glutamine measurement.

We found a significant difference between the glutamate levels in the ALF and FPSA groups from 4 hours to 11 hours after surgery; the change in glutamate in the FPSA group resembled the change in the L/P ratio. However, there was only a slight difference between the ALF and SHAM groups; glutamate was higher in the ALF group, but only significantly so between 4 hours and 9 hours after surgery. Moreover, there was no significant difference between the FPSA and the SHAM group. These results exclude glutamate as a reliable marker in monitoring the efficacy of the treatment of ALF.

Extracellular brain glucose was higher in the SHAM group than in the ALF or FPSA groups. Although uncommon in ALF, similar findings have been reported before [26]. Nevertheless, when we compared the changes in extracellular brain glucose from baseline, there were no significant differences between the groups, suggesting that FPSA does not influence cerebral extracellular glucose levels.

Plasma ammonia levels were significantly higher in both the ALF and FPSA groups than the SHAM group, and the difference between the FPSA and ALF groups was also significant. As there is a relatively wide range of accepted physiological values for plasma ammonia, we also compared the values relative to baseline. This comparison showed a significant difference between the FPSA and ALF groups as early as 4 hours after surgery, demonstrating the efficacy of FPSA therapy.

## Conclusions

FPSA treatment significantly attenuates the development of intracranial hypertension and decreases plasma ammonia levels, both of which are fundamental to the pathophysiological processes that underpin ALF.

Cerebral microdialysis is a valuable and promising technique; the L/P ratio and intracerebral glutamin reflected the beneficial effects of FPSA treatment on cerebral metabolism more quickly than ICP in our experimental model of ALF.

Glutamate did not show significant changes, and thus its clinical use cannot presently be recommended. However, the role of glutamine as a clinical marker of the efficacy of treatment warrants further investigation.

## Competing interests

The authors declare that they do not have anything to disclose regarding conflicts of interest with respect to this manuscript.

Supported by grant NT 11463 of the Ministry of Health and IP ZRO MO1012, Czech Republic.

## Authors’ contributions

JP and MR generated the hypothesis, and conceived and designed the study. JP, EL, OR, TP and EK contributed to data collection. JP undertook data interpretation and statistical analyses. JP drafted the manuscript. EL, OR, TP, EK and MR revised the manuscript. All authors read and approved the final manuscript.

## Pre-publication history

The pre-publication history for this paper can be accessed here:

http://www.biomedcentral.com/1471-230X/13/98/prepub

## References

[B1] Detry O De Roover A Honoré P Meurisse M Brain edema and intracranial hypertension in fulminant failure: pathophysiology and management World J Gastroenterol 2006 12 7405 7412 17167826 10.3748/wjg.v12.i46.7405 PMC4087583

[B2] Bismuth H Samuel D Castaing D Adam R Saliba F Johann M Orthotopic liver transplantation in fulminant and subfulminant hepatitis Ann Surg 1995 222 109 119 10.1097/00000658-199508000-00002 7639578 PMC1234768

[B3] Ryska M Laszikova E Pantoflicek T Ryska O Prazak J Fractionated plasma separation and adsorption significantly decreases intracranial pressure in acute liver failure: experimental study Eur Surg Res 2009 42 230 235 10.1159/000208790 19279388

[B4] Bjerring FN Hauerberg J Jorgensen L Frederiksen HJ Tofteng F Hansen BA Brain hypoxanthine concentration correlates to lactate/pyruvate ratio but not intracranial pressure in patients with acute liver failure J Hepatol 2010 53 1054 1058 10.1016/j.jhep.2010.05.032 20800925

[B5] Bjerring PN Hauerberg J Frederiksen HJ Nielsen HB Clemmesen JO Larsen FS The effect of fractionated plasma separation and absorption on cerebral amino acid metabolism and oxidative metabolism during acute liver failure J Hepatol 2012 57 774 779 10.1016/j.jhep.2012.06.004 22691571

[B6] Ladurner R Hochleitner B Schneeberger S Barnas U Krismer A Kleinsasser A Extended liver resection and hepatic ischemia in pigs: a new, potentially reversible model to induce acute liver failure and study artificial liver support systems Eur Surg Res 2005 37 6 365 369 10.1159/000090338 16465062

[B7] Rifai K Ernst T Kretschmer U Hafer C Haller H Manns MP The Prometheus® device for extracorporeal support of combined liver and renal failure Blood Purif 2005 23 4 298 202 10.1159/000086552 15980619

[B8] Reinstrup P Stahl N Mellergard P Uski T Ungerstedt U Nordstrom CH Intracerebral microdialysis in clinical practice: baseline values for chemical markers during wakefulness, anesthesia, and neurosurgery Neurosurgery 2000 47 3 701 709 10981758 10.1097/00006123-200009000-00035

[B9] Stahl N Mellergard P Hallstrom A Ungerstedt U Nordstrom CH Intracerebral microdialysis and bedside biochemical analysis in patients with fatal traumatic brain lesions Acta Anaesthesiol Scand 2001 45 8 977 985 10.1034/j.1399-6576.2001.450810.x 11576049

[B10] Rose C Michalak A Pannunzio M Chatauret N Rambaldi A Butterworth RF Mild hypothermia delays the onset of coma and prevents brain edema and extracellular brain glutamate accumulation in rats with acute liver failure Hepatology 2000 31 4 872 877 10.1053/he.2000.5923 10733542

[B11] Falkenhagen D Strobl W Vogt G Schrefl A Linsberger I Gerner FJ Fractionated plasma separation and absorption system: a novel system for blood purification to remove albumin bound substances Artif Organs 1999 23 1 81 86 10.1046/j.1525-1594.1999.06292.x 9950184

[B12] Kramer DJ Canabal JM Arasi LC Application of intensive care medicine principles in the management of acute liver failure patient Liver Transpl 2008 14 suppl 2 S85 S89 18825685 10.1002/lt.21649

[B13] Auzinger G Wendon J Intensive care management of acute liver failure Curr Opin Crit Care 2008 14 2 179 188 10.1097/MCC.0b013e3282f6a450 18388681

[B14] Bjerring PN Eefsen M Hansen BA Larsen FS The brain in acute liver failure. A tortuous path from hyperammonemia to cerebral edema Metab Brain Dis 2009 24 5 14 10.1007/s11011-008-9116-3 19050999

[B15] Dethloff TJ Knudsen GM Larsen FS Cerebral blood flow autoregulation in experimental liver failure J Cereb Blood Flow Metab 2008 28 5 916 926 10.1038/sj.jcbfm.9600589 18059432

[B16] Ungerstedt U Microdialysis–principles and applications for studies in animals and man J Intern Med 1991 230 4 365 373 10.1111/j.1365-2796.1991.tb00459.x 1919432

[B17] Hillered L Persson L Neurochemical monitoring of the acutely injured human brain Scand J Clin Lab Invest Suppl 1999 229 9 18 10097285 10.1080/00365519950185904

[B18] Bellander B-M Cantais E Enblad P Hutchinson P Nordström C-H Robertson C Sahuquillo J Smith M Stocchetti N Ungerstedt U Unterberg A Vidiendal Olsen N Consensus meeting on microdialysis in neurointensive care Intensive Care Medcine 2004 30 2166 2169 10.1007/s00134-004-2461-8 15549254

[B19] Ungerstedt U Continuous Monitoring of Organ Chemistry — a Paradigm Shift in Management of Intensive Care - Anaesthesia, Pain, Intensive Care and Emergency A.P.I.C.E 2008 29 4423785706

[B20] Tofteng F Hauerberg J Hansen BA Pedersen CB Jørgensen L Larsen FS Persistent arterial hyperammonemia increases the concentration of glutamine and alanine in the brain and correlates with intracranial pressure in patients with fulminant hepatic failure J Cereb Blood Flow Metab 2006 26 1 21 27 10.1038/sj.jcbfm.9600168 15959460

[B21] Martinez-Hernandez A Bell KP Norenberg MD Glutamine synthetase: glial localization in brain Science 1977 195 4284 1356 1358 10.1126/science.14400 14400

[B22] Brusilow SW Traystman R Hepatic encephalopathy N Engl J Med 1986 314 12 786 787 15603053

[B23] Albrecht J Norenberg MD Glutamine: a Trojan horse in ammonia neurotoxicity Hepatology 2006 44 4 788 794 10.1002/hep.21357 17006913

[B24] Norenberg MD Oxidative and nitrosative stress in ammonia neurotoxicity Hepatology 2003 37 2 245 248 10.1053/jhep.2003.50087 12540772

[B25] Bjerring PN Hauerberg J Frederiksen HJ Jorgensen L Hansen BA Tofteng F Cerebral glutamine concentration and lactate–pyruvate ratio in patients with acute liver failure Neurocrit Care 2008 9 1 3 7 10.1007/s12028-008-9060-4 18250976

[B26] Hutchinson PJ Gimson A Al-Rawi PG O'Connell MT Czosnyka M Menon DK Microdialysis in the management of hepatic encephalopathy Neurocrit Care 2006 5 3 202 205 10.1385/NCC:5:3:202 17290089

